# *PRPH2* mutation c.582-1G>A causing adult-onset macular dystrophy with a benign concentric annular macular dystrophy phenotype in a family

**DOI:** 10.5935/0004-2749.2021-0478

**Published:** 2023-03-08

**Authors:** Karina Fernández-Berdasco, José Galvez-Olortegui, Sussan’s Pamela Guillén-Lozada, Noelia García González, Joaquín Castro-Navarro

**Affiliations:** 1 Ophthalmology Department, Hospital Universitario Central de Asturias, Oviedo, Spain; 2 Evidence Based Ophthalmology Unit (Oftalmoevidencia), Scientia Clinical and Epidemiological Research Institute, Trujillo, Peru; 3 Genetics Department, Hospital Universitario Central de Asturias, Oviedo, Spain

**Keywords:** PRPH2 gene, Mutation, Macular degeneration, Stargardt disease, Gene PRPH2, Mutação, Degeneração macular, Doença de Stargardt

## Abstract

The peripherin gene (*PRPH2*) mutation is associated with photoreceptor cell dysfunction as well as in several inherited retinal dystrophies. The *PRPH2* mutation c.582-1G>A is a rare variant reported in retinitis pigmentosa and pattern dystrophy. Here Case 1 was of a 54-year-old woman with bilateral atrophy of the perifoveal retinal pigmentary epithelium and choriocapillaris with central foveolar respect. Autofluorescence and fluorescein angiography revealed perifoveal atrophy of the retinal pigmentary epithelium with an annular window effect without the “dark choroid” sign. Case 2 (mother of Case 1) presented with extensive atrophy of the retinal pigmentary epithelium and choriocapillaris. *PRPH2* was evaluated and the c.582-1G>A mutation was identified in heterozygosity. An advanced adult-onset benign concentric annular macular dystrophy diagnosis was thereby proposed. The c.582-1G>A mutation is poorly known and not present in all common genomic databases. This case report is the first one to report a c.582-1G>A mutation associated with benign concentric annular macular dystrophy.

## Abstract (pt)

Mutações do gene da periferina (PRPH2) estão associadas à disfunção das células fotorreceptoras e estão envolvidas em várias distrofias retinianas hereditárias. A mutação c.582-1G>A do gene PRPH2 é uma variante rara, relatada na retinite pigmentosa e nas distrofias em padrão. O caso 1 foi de uma mulher de 54 anos com atrofia bilateral do epitélio pigmentar da retina perifoveal e da coriocapilar, com acometimento foveolar central. A autofluorescência e a angiofluoresceinografia revelaram atrofia perifoveal do epitélio pigmentar da retina, com efeito de janela anular, sem o sinal da “coroide escura”. O caso 2 (mãe) apresentava extensa atrofia do epitélio pigmentar da retina e da coriocapilar. Foi feito um estudo do gene PRPH2, que identificou a mutação c.582-1G>A em heterozigose. Foi proposto um diagnóstico de distrofia macular anular concêntrica benigna de início adulto em estágio avançado. A mutação c.582-1G>A é pouco conhecida e não está presente em todos os bancos de dados genômicos usuais. Este é o primeiro relato de caso publicado de uma mutação c.582-1G>A associada à distrofia macular anular concêntrica benigna.

## INTRODUCTION

Benign concentric annular macular dystrophy (BCAMD) is a rare retinal disease that was first described by Deutman in 1974^(1)^. It is characterized by the presence of bull’s-eye maculopathy with annular atrophy of the pigment epithelium in the perifoveal retina with central respect^(1-5)^. The differential diagnosis for BCAMD includes hydroxychloroquine maculopathy, age-related macular degeneration, cone-rod dystrophy, pattern dystrophy (PD), and Stargardt disease^(5)^. Mutations in the interphotoreceptor matrix proteoglycan 1 (IMPG1), cone-rod homeobox (CRX), prominin 1 (PROM-1), and peripherin (PRPH2) are associated with BCAMD^(2-5)^.

Peripherin gene (*PRPH2*) is involved in diverse inherited retinal dystrophies, such as retinitis pigmentosa (RP), Best’s Vitelliform macular dystrophy, cone-rod, and PD^(6)^. PRPH2 (OMIM 179605) encodes a cell-surface glycoprotein related to the proper formation and maintenance of the outer segment of photoreceptors^(6)^. Mutations in the *PRPH2* are associated with the misfunctioning of the photoreceptor cells with the accumulation of lipofuscin and the consequent RPE cell damage. PRPH2 mutations have an autosomal dominant inheritance, although several autosomal recessive variants have been reported so far^(6)^.

This report describes the first published case of adult-onset macular dystrophy with a BCAMD phenotype associated with the c.582-1G>A mutation in *PRPH2*. The c.582-1G>A mutation is a recently discovered mutation in a splice site considered to be likely pathogenic and identified using molecular inversion probes (MIPs)^(6-8)^. MIPs are a novel and emerging sequencing approach that allows high specificity, a smaller amount of DNA sample, and high concordance and reproducibility when compared with conventional sequencing techniques, which translates to lower costs^(7)^.

## CASE REPORT

**Figure 1:**
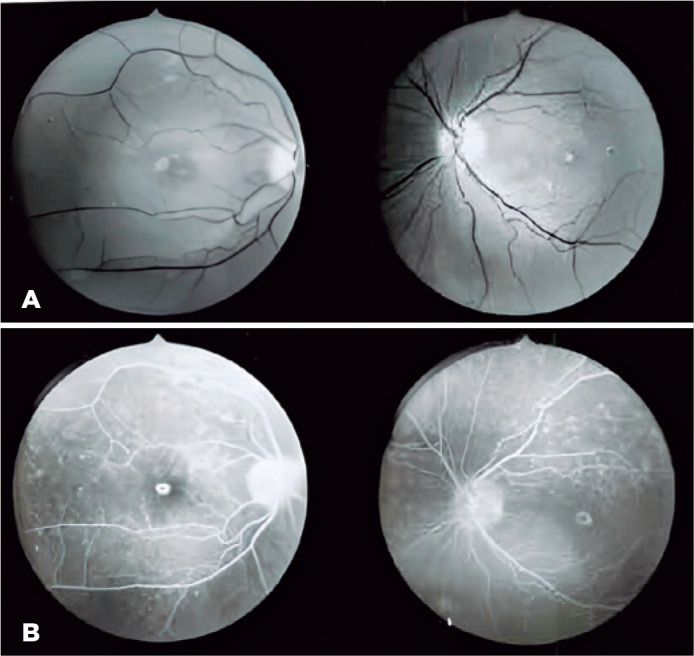
(A) Red-free light fundus photography showing initial perifoveal pigmentary alterations in the RPE. (B) Fluorescein angiography with characteristic bull’s eye-like hyperfluorescent perifoveal ring due to the “window” effect.

A 54-year-old woman (Case 1) was referred to the retina unit of our hospital under suspicion of Stargardt disease. Twenty years ago (at the age of 30 years), she was evaluated for macular alteration with annular perifoveal atrophy of the RPE in both eyes (hyperfluorescent on FA) (Figure 1). Maculopathy was not related to a pharmacological cause and she showed no history of chloroquine or hydroxychloroquine treatment. With respect to family history, she referred to no previously studied visual disability in her mother.

**Figure 2:**
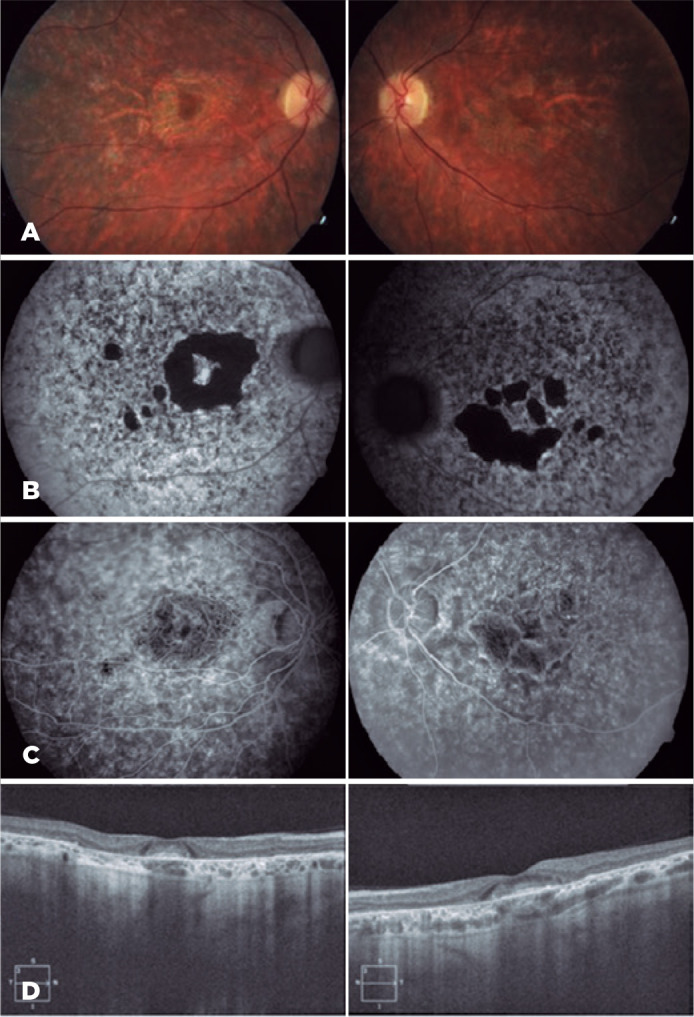
(A) Fundus showing atrophy of the perifoveal RPE and choriocapillary, with central foveolar respect, symmetrically in both the eyes. (B) FFA images showing high hypoautofluorescence, confirming the absence of RPE and pigmentary accumulation in the peripheral retina, thereby exhibiting a low AF signal. (C) FA with a “window defect” because of the absence of RPE and choriocapillary without the “dark choroid” sign. (D) SD-OCT showing moderate retinochoroidal atrophy with central subfoveal accumulation in both the eyes.

**Figure 3:**
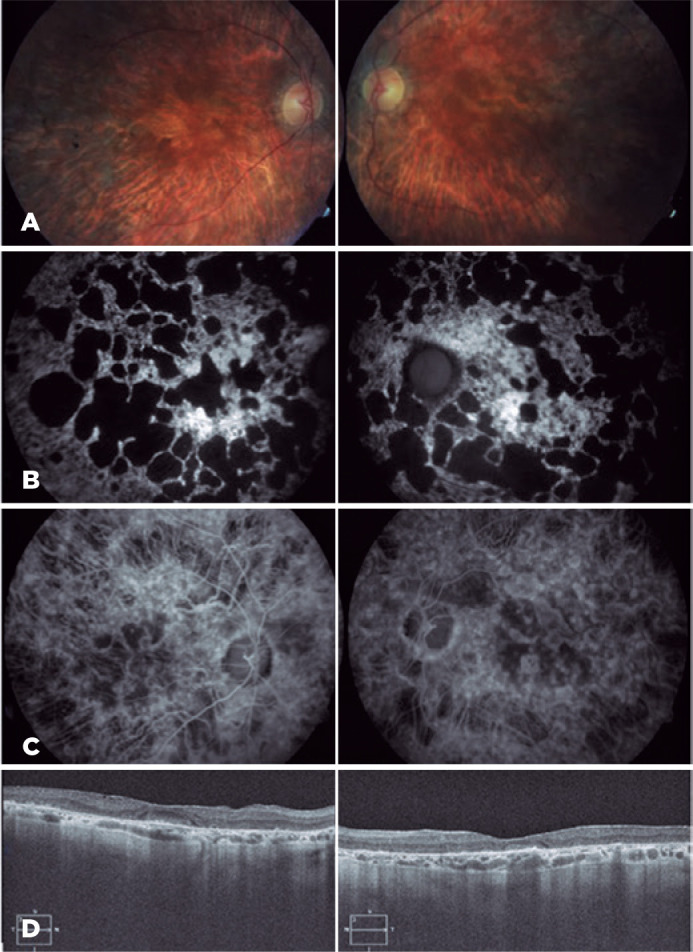
(A) Fundus with extensive atrophy of the RPE and choriocapillaris in perifoveal patches with central respect in the posterior pole and equator. (B) FFA showing severe RPE atrophy. (C) FA with external retinal and choriocapillary atrophy and preserved Haller’s layer. (D) SD-OCT with choriocapillary atrophy and epiretinal membrane.

Her visual acuity (BCVA) was 20/20 in the right eye and 16/20 in the left eye. Anterior biomicroscopy and intraocular pressure findings were normal. Fundus examination showed advanced perifoveal RPE and choriocapillary atrophy, with central foveolar respect, symmetrically in both the eyes in addition to the pigmentary accumulations in the peripheral retina without optic nerve pallor, bone spicules, or attenuated vessels (Figure 2). FA and fluorescein angiography autofluorescence (FFA) were performed, with central hypoautofluorescence confirming the symmetrical absence of RPE and choriocapillaris and an annular hyperfluorescent bull’s-like perifoveal ring without the “dark choroid” sign characteristic of Stargardt disease. Spectral-domain optical coherence tomography (SD-OCT) showed moderate retinochoroidal atrophy with central subfoveal accumulation in both eyes (Figure 3).

Case 1’mother (an 82-year-old woman) was also examined at the same visit (Case 2). She reported progressive visual acuity loss, which was more pronounced in recent years. Her BCVA was 10/20 in both the eyes and fundus examination revealed extensive atrophy of the RPE and choriocapillaris in perifoveal patches with central respect, predominantly in the posterior pole and the equator region (Figure 3).

FFA and FA findings were identical to those of her daughter, with a greater degree of progression, consisting of external retinal and choriocapillary atrophy and preserved Haller’s layer. SD-OCT showed atrophy and subfoveal accumulation associated with an epiretinal membrane (Figure 3).

Considering these findings, as well as the BCVA and patient age, the condition appeared to correspond to adult-onset macular dystrophy with a BCAMD phenotype for both the patients at an advanced stage. An autosomal dominant inheritance disease was accordingly suspected, and, therefore, a genetic study was performed. The exonic sequence and the flanking regions of *PRPH2* were analyzed a change was identified in heterozygosity c.582-1G>A (p.-), which was classified as pathogenic and compatible with the clinical diagnosis.

**Figure 4:**
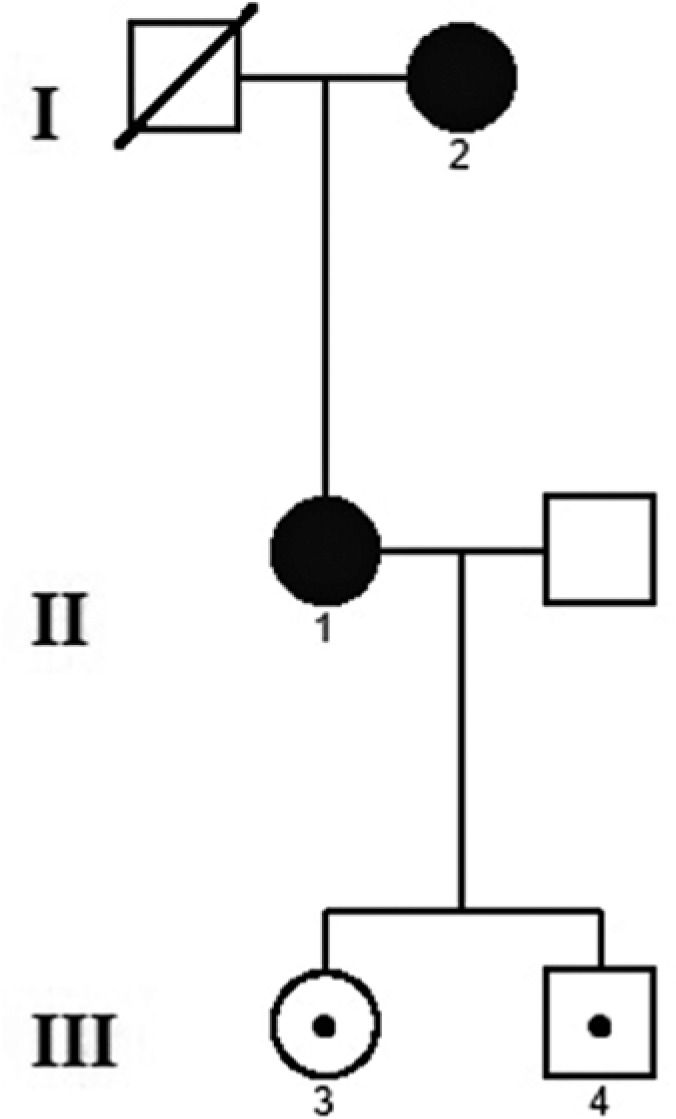
Pedigree chart of the family with the c.582-1G>A mutation in *PRPH2*.

Case 1 has a 22-year-old daughter who has difficulty with night vision (Case 3) and a 19-year-old son (Case 4), who had also undergone a genetic study confirming their status as mutation carriers. A complete ophthalmological examination of Cases 3 and 4 was performed, but no evidence of maculopathy or retinal disorder was determined. For further comprehension, a family tree is illustrated in figure 4.

## DISCUSSION

The c.582-1G>A (p.-) mutation is rare and was first described in 2015 by Fernández-Sanz José et al. through a next-generation sequencing (NGS) study based on a Spanish cohort of autosomal dominant RP^(7,8)^. The presence of this mutation has also been documented in patients with an overlap phenotype between STFD1 and PD^(6,9,10)^. There is evidence of *PRPH2* mutations in Bull’s eye maculopathy^(5)^; however, based on the available evidence, this is the first report of a PRPH2/RDS c.582-1G>A mutation associated with BCAMD.

A characteristic autofluorescence pattern of *PRPH2* mutation has been described in BCAMD, which involves central hyperautofluorescence with a marginal hyper and hypoautofluorescent mottling at the early stages^(5)^. In our case, FFA was not performed at the initial stages (Figure 1), but an alteration of the RPE surrounding the annular areas of atrophy with the characteristic hyperautofluorescent speckled appearance was noticeable (Figure 4). The main limitation of this work is the late diagnosis of macular dystrophy in the case family; therefore, its correct classification is a diagnostic challenge.

Genetic studies play an essential role in retinal dystrophy diagnosis and allow the establishment of the type of inheritance and genetic advice. NGS for single-nucleotide variants and copy number variation screening are less laborious and expensive relative to conventional techniques and they allow targeting of a greater number of variants irrespective of the phenotype or the inheritance type.

Despite the development of new techniques and genetic tools, the diagnosis and classification of macular dystrophy remain difficult. Although genetic testing is useful for identifying retinal dystrophy, clinical examination and multimodal imaging tests are used to establish a diagnosis. Considering the clinical variability between individuals carrying the same genetic mutation, further studies are warranted to understand the etiopathogenesis of macular dystrophies as well as to develop a personalized medicine approach in the future.
